# Comparison between laparotomy first versus angiographic embolization first in patients with pelvic fracture and hemoperitoneum: a nationwide observational study from the Japan Trauma Data Bank

**DOI:** 10.1186/1757-7241-21-82

**Published:** 2013-12-03

**Authors:** Morihiro Katsura, Shin Yamazaki, Shingo Fukuma, Kazuhide Matsushima, Toshimitsu Yamashiro, Shunichi Fukuhara

**Affiliations:** 1 Department of Surgery, Okinawa Prefectural Hokubu Hospital, Okinawa, Japan; 2 Department of Healthcare Epidemiology, Kyoto University Graduate School of Medicine and Public Health, Yoshida Konoe-cho, Sakyo-ku, Kyoto, Japan; 3 Department of Surgery, University of Southern California, Los Angeles, CA, USA

**Keywords:** Pelvic fracture, Hemoperitoneum, Laparotomy, Angiographic embolization

## Abstract

**Background:**

A common dilemma in the management of pelvic fractures is recognizing the presence of associated abdominal injury. The purpose of this study was to determine the association between initial therapeutic intervention (laparotomy or transcatheter arterial embolization (TAE)) and mortality.

**Methods:**

This was a cohort study using the Japan Trauma Data Bank between 2004 and 2010, including blunt trauma patients with pelvic fractures and positive Focused Assessment with Sonography in Trauma (FAST) results. Eligible patients were restricted to those who underwent laparotomy or TAE/angiography as the initial therapeutic intervention. Crude and adjusted odds ratio (AOR) for in-hospital mortality were compared between the laparotomy first and TAE first groups (reference group). Multiple logistic regression analysis and propensity score adjusted analysis were used to adjust for clinically relevant confounders, including the severity of injury.

**Results:**

Of the 317 participants, 123 patients underwent laparotomy first and 194 patients underwent TAE first. The two groups were similar in terms of age, although the laparotomy first group had higher mean Injury Severity Scores (ISS) and higher mean scores based on the abdominal Abbreviated Injury Scale (AIS), as well as lower mean pelvic AIS and systolic blood pressure (SBP). Half of the patients who were hypotensive (SBP < 90 mmHg) on arrival underwent TAE first. The laparotomy first group had a significantly higher crude in-hospital mortality (41% vs. 27%; P < 0.01). After adjusting for confounders, the choice of initial therapeutic intervention did not affect the in-hospital mortality (AOR, 1.20; 95% Confidence Interval (CI), 0.61-2.39). Even in the limited subgroup of hypotensive patients (SBP 66–89 mmHg and SBP < 65 mmHg subgroup), the effect was similar (AOR, 1.50; 95% CI, 0.56-4.05 and AOR, 1.05; 95% CI, 0.44-3.03).

**Conclusions:**

In Japan, laparotomy and TAE are equally chosen as the initial therapeutic intervention regardless of hemodynamic status. No significant difference was seen between the laparotomy first and TAE first groups regarding in-hospital mortality.

## Background

Despite advances in trauma care, the appropriate management of hemorrhage due to pelvic fractures and associated abdominal injuries remains a big challenge for general surgeons [1-4]. The pelvic ring is composed of two stiff coxal bones, the sacrum and their supporting strong ligaments. Pelvic fractures usually occur with high-energy blunt trauma, such as occurs in motor vehicle crashes or falls, causing multiple life-threatening injuries to the organs of the entire body [3]. In a preceding study, isolated fracture of the pelvis appeared in only 14% of patients, most of who suffered from additional associated injuries in other organ systems^1^. The overall frequency of additional intra-abdominal injuries in patients with unstable pelvic fractures is reportedly as high as 67% [1-4]. However, it is quite difficult to decide the precise management priorities in patients with both retroperitoneal bleeding from pelvic fractures and free bleeding into the intraperitoneal space.

Some review articles and practice management guidelines for pelvic trauma patients have been published in the United States and Europe [5,6]. These articles have recommended that hemodynamically unstable patients with pelvic fractures and positive Focused Assessment with Sonography in Trauma (FAST) results should proceed for immediate exploratory laparotomy [5,6]. A number of small retrospective studies have described the practice patterns and outcomes in patients with pelvic fractures and hemoperitoneum [1,7-9]. However, evidence to support these recommendations has yet to be found, and thus the clinical dilemma about whether laparotomy or transcatheter arterial embolization (TAE) should be the initial therapeutic intervention in these difficult-to-manage patients has yet to be resolved.

Since the sequence of these interventions varies according to institutional resources and policy, we suppose that many practice variations exist in each country and each institution. To our knowledge, no large comparative analytic study has documented the association between initial therapeutic intervention (laparotomy or TAE) and mortality, after taking hemodynamic stability and the severity of injury in pelvic trauma patients into consideration. The purpose of this study was to determine the association between initial therapeutic intervention (laparotomy or TAE) and in-hospital mortality.

## Methods

### Study design and data source

We conducted a historical cohort study using data derived from the prospectively maintained Japan Trauma Data Bank (JTDB) during the years 2004 through 2010. The JTDB was started in 2003 by the Japanese Association for Trauma Surgery (Trauma Registry Committee) and the Japanese Association for Acute Medicine (Committee for Clinical Care Evaluation). The Association for Japan Trauma Care Research (JTCR) assumed the lead role in training the AIS-certified trauma registry coders. The JTDB represents a large national repository of trauma patients. Data are continuously inputted into a web-based data server from 147 major, voluntarily participating emergency hospitals in Japan in 2011. The registry records contain each patient’s demographic data [age, gender, vital signs on-scene and at presentation at the emergency department (ED)]; mechanism of injury; pre-existing medical conditions according to the International Classification of Diseases (ICD-10); diagnostic, operative, and interventional information; injury severity; and patient disposition [10-12]. Diagnosis of injury is recorded according to the Abbreviated Injury Scale (AIS) using AIS 90 Update 98. The severity of anatomic injuries is evaluated using the Injury Severity Score (ISS) and the severity of physiological injuries is evaluated using the Revised Trauma Score (RTS). Probability of survival (Ps) was calculated using these data and Trauma and the Injury Severity Score (TRISS) method. The JTDB also contains data about FAST, which detects free intraperitoneal fluid and pericardial effusion. This study received full approval of the ethics committee of Kyoto University.

### Patient selection and definitions

This study included blunt trauma patients who had both pelvic fractures and positive FAST results. Eligible patients included those who underwent either laparotomy or TAE/angiography as the initial therapeutic intervention. To control for potential confounders in the design stage of the study, patients with penetrating trauma were excluded from analysis. To minimize concern about other organ injuries as potential confounders, we also excluded patients with unsalvageable severe head injury (head AIS ≥ 5) and those who underwent a different initial therapeutic intervention, such as craniotomy/craterization, thoracotomy, including both resuscitative thoracotomy and pericardiocentesis/pericardiotomy in the ED, bone fixation surgery, other types of surgery, non-surgical management and non-classifiable cases. We also excluded patients who were dead on arrival (DOA). We defined patients as DOA if their systolic blood pressure (SBP), respiratory rate and Glasgow Coma Scale (GCS) scores were at the minimum values on arrival [13]. Hypotension was defined as SBP < 90 mmHg.

The primary outcome of interest was in-hospital mortality. The secondary outcome of interest was mortality within 24 hours of hospital admission. The primary independent variable was the initial therapeutic intervention (laparotomy or TAE/angiography).

### Statistical analysis

We performed a descriptive analysis of our dependent variables. Patients were then divided into two groups based on whether they underwent laparotomy (laparotomy first group) or TAE/angiography (TAE first group) as the initial therapeutic intervention. Data are summarized as mean ± standard deviation (SD) or number and percentage (%). We performed a descriptive data analysis comparing patient demographics between the laparotomy first and TAE first groups, using χ^2^ analysis for categorical variables and Student’s t-test or Mann–Whitney U test for continuous variables.

We conducted an unadjusted analysis that included a comparison of in-hospital mortality and mortality within 24 hours between the laparotomy first and TAE first groups (reference group). The results were presented as risk ratio (RR) and odds ratio (OR) with 95% confidence intervals (CI). Significant differences were found between the two groups based on the known risk factors for death, including the severity of injuries; therefore, we performed multivariable analysis. To adjust for pretreatment imbalances of background clinical characteristics, we used two statistical approaches. First, a multiple logistic regression model (model 1) was used to analyze associations between initial therapeutic intervention (laparotomy or TAE) and in-hospital mortality. Covariates in this regression model, in addition to initial therapeutic intervention variables, included age, gender, number of comorbidities, SBP and GCS score in the ED, and both ISS and AIS of major bodily injuries (pelvic AIS, head AIS, thoracic AIS and abdominal AIS). Second, we used a propensity score methodology (model 2) to calculate the propensity score, i.e. the conditional probability of undergoing laparotomy as the initial therapeutic intervention, given all the potential confounders measured. The propensity score was calculated through a multiple logistic regression model using ‘undergoing laparotomy’ as the dependent variable and the same confounders listed in model 1 as independent variables. The propensity score was then used to perform multiple logistic regression analysis of only independent variables. In addition to these analyses, we also performed subgroup analysis according to clinically relevant confounders, as indicated. The same unadjusted analysis and multiple logistic regression analysis was rerun on the following subgroups of trauma patients: (1) SBP (≤65 mmHg, 66–89 mmHg or ≥90 mmHg), (2) pelvic AIS (≤3 or ≥4), and (3) abdominal AIS (≤3 or ≥4).

We chose the simple approach of eliminating patients with missing data about covariates and discharge disposition because the proportion of missing data was small. A multiple imputation approach using chained equations was also used to account for missing covariates as a sensitivity analysis. Statistical analyses were two-sided, with a P value of 0.05 considered to indicate statistical significance. All analyses were performed using Stata/SE 11 (StataCorp, College Station, TX, USA).

## Results

### Study participants and baseline characteristics

**Figure 1:**
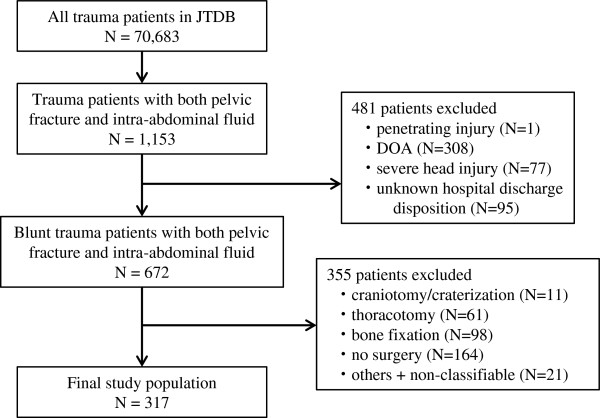
**Selection process for the study population.** DOA, dead on arrival; JTDB, Japan Trauma Data Bank.

During the study period, 147 emergency hospitals submitted data on 70,683 patients to the JTDB. Of the 70,683 patients, 1,153 were diagnosed with both pelvic fractures and positive FAST results. Of these 1,153 eligible patients, 481 patients were excluded because of penetrating injury (1 patient), DOA (308 patients), head AIS ≥ 5 (77 patients) and unknown hospital discharge disposition (95 patients). Of the remaining 672 patients, 355 patients were excluded because they underwent an initial therapeutic intervention other than laparotomy or TAE. The interventions performed in these excluded patients and their numbers (proportion of death (%)) were craniotomy/craterization in 11 patients (73%), thoracotomy in 61 patients (77%), bone fixation surgery in 98 patients (5%), no operation in 164 patients (37%) and other type of operation or non-classifiable in 21 patients (48%). A total of 317 patients from the 87 institutions that submitted data were analyzed in this study. The process used to select participants from the database is shown in Figure 1.

Baseline demographics and clinical characteristics of all participants in this study are summarized in Table 1. The overall mean age of participants was 48.8 years, 58% were men and 43% were found to have one or more comorbidities. Upon arrival in the ED, 51% of patients were hypotensive (SBP < 90 mmHg). With regard to injury severity, the mean pelvic AIS was 3.5, mean abdominal AIS was 2.5, mean ISS was 37.4, mean RTS was 6.3 and mean Ps, according to the TRISS method, was 0.63. Associated abdominal organ injuries of the liver (31%), spleen (22%), kidney (14%), mesentery (11%), bladder (8%) and bowel perforation (7%) were also present in some of the patients. No major differences in background demographics were found between eligible patients and those whose outcome data were missing.

**Table 1:** Baseline demographics and clinical characteristics of the study subjects

		**Number (percent)**	**Laparotomy first**	**TAE first**	***P***
		**(Total number = 317)**	**(N = 123)**	**(N = 194)**	
**Patient characteristics**					
Age	years (mean ± SD)	48.8 ± 22.5	48.7 ± 21.3	48.9 ± 23.3	0.955
	≤25	67 (21%)	24 (20%)	43 (22%)	
	26-49	91 (29%)	36 (29%)	55 (28%)	
	50-64	64 (20%)	34 (28%)	30 (15%)	
	≥65	94 (30%)	28 (23%)	66 (34%)	
Gender					<0.001*
	male	185 (58%)	86 (70%)	99 (51%)	
	female	132 (42%)	37 (30%)	95 (49%)	
No. of comorbidities					0.346
	0	181 (57%)	74 (60%)	107 (55%)	
	1	106 (33%)	39 (32%)	67 (35%)	
	≥2	30 (10%)	10 (8%)	20 (10%)	
**Pre-hospital**					
	artificial respiration	14 (4%)	10 (8%)	4 (2%)	0.010*
	prehospital IV	31 (10%)	15 (12%)	16 (8%)	0.249
**In-hospital**					
SBP	mmHg (mean ± SD)	91 ± 33	84 ± 32	97 ± 33	<0.001*
	≤65	67 (21%)	35 (28%)	32 (16%)	
	66-89	96 (30%)	46 (37%)	50 (26%)	
	≥90	151 (48%)	42 (34%)	109 (56%)	
HR	beat/min (mean ± SD)	104 ± 28	104 ± 30	104 ± 26	0.927
BT	°C (mean ± SD)	35.8 ± 1.2	35.6 ± 1.0	35.9 ± 1.2	0.063
GCS score	mean ± SD	11.7 ± 4.0	11.1 ± 4.5	12.1 ± 3.7	0.035*
(total)	< 9	69 (23%)	36 (31%)	33 (18%)	
	9-13	76 (25%)	25 (21%)	51 (27%)	
	> 13	160 (52%)	57 (48%)	103 (55%)	
**Severity of injuries**					
ISS	mean ± SD	37.4 ± 13.9	38.5 ± 12.9	33.8 ± 13.9	0.027*
	< 26	80 (25%)	19 (15%)	61 (31%)	
	26-35	86 (27%)	35 (28%)	51 (26%)	
	36-45	76 (24%)	31 (25%)	45 (23%)	
	> 45	75 (24%)	38 (31%)	37 (19%)	
RTS	mean ± SD	6.3 ± 1.6	5.8 ± 1.8	6.6 ± 1.4	<0.001*
Ps(TRISS)	mean ± SD	0.63 ± 0.32	0.53 ± 0.34	0.68 ± 0.30	<0.001*
AIS					
	Pelvic fracture	3.5 ± 1.3	3.3 ± 1.2	3.6 ± 1.3	0.025*
	Head	0.9 ± 1.5	1.0 ± 1.5	0.9 ± 1.5	0.863
	Face	0.3 ± 0.6	0.3 ± 0.6	0.2 ± 0.6	0.462
	Thorax	2.4 ± 1.9	2.5 ± 1.9	2.3 ± 1.9	0.374
	Abdomen	2.5 ± 1.6	3.4 ± 1.2	2.0 ± 1.6	<0.001*
	Upper extremity	0.7 ± 1.0	0.7 ± 1.0	0.7 ± 1.0	0.997
**Associated injury**					
	Liver	99 (31%)	45 (37%)	54 (28%)	0.101
	Spleen	70 (22%)	29 (24%)	41 (21%)	0.609
	Kidney	45 (14%)	21 (17%)	24 (12%)	0.242
	Bladder	25 (8%)	12 (10%)	13 (7%)	0.325
	Bowel perforation	21 (7%)	17 (14%)	4 (2%)	<0.001*
	Mesenteric	34 (11%)	27 (22%)	7 (4%)	<0.001*

AIS, abbreviated injury scale; BT, body temperature; GCS, Glasgow coma scale; HR, heart rate; ISS, injury severity score; IV, intravascular; Ps, probability of survival; RTS, revised trauma score; SBP, systolic blood pressure; SD, standard deviation; TAE, transcatheter arterial embolization; TRISS, trauma and injury severity score.

*p values significant at p < 0.05.

Of the 317 participants, 123 underwent laparotomy (laparotomy first group) and 194 underwent TAE/angiography (TAE first group) as the initial therapeutic intervention. The laparotomy first group had a higher proportion of men, a higher mean ISS, and a higher mean abdominal AIS score than the TAE first group. The laparotomy group had a lower mean GCS score and was more likely to present with a lower mean SBP. On the other hand, the TAE group had a higher mean pelvic AIS score and showed better Ps than the laparotomy group. Approximately half (50%) of the patients who were hypotensive in the ED underwent TAE as the initial therapeutic intervention.

### Association between initial therapeutic intervention and in-hospital mortality

Results of unadjusted comparisons of mortality between the two groups are shown in Table 2. Using the TAE first group as a reference, the laparotomy first group had both a significantly higher unadjusted, crude in-hospital mortality rate (RR, 1.52; 95% CI, 1.11-2.08 and OR, 1.87; 95% CI, 1.12-3.11) and a higher mortality rate within 24 hours (RR, 1.71; 95% CI, 1.16-2.51 and OR, 2.04; 95% CI, 1.17-3.56).

**Table 2:** Unadjusted comparison of mortality in laparotomy first versus TAE first cases

**Outcome**		**All patients**	**Laparotomy first**	**TAE first**	**RR**	**95% CI**
		**N = 317**	**N = 123**	**N = 194**		
**Death within 24 hr**	(number [%])	77 (24%)	40 (33%)	37 (19%)	1.71	1.16-2.51*
**Death in hospital**	(number [%])	102 (32%)	50 (41%)	52 (27%)	1.52	1.11-2.08*

CI, confidence intervals; RR, risk ratio; TAE, transcatheter arterial embolization.

*p values significant at (p < 0.05).

Multivariable analyses were then performed to determine the association between initial therapeutic intervention and in-hospital mortality (Table 3). To adjust for potential confounders, we created a multiple logistic regression model (model 1) using patients’ characteristics, vital signs in the ED, and severity of injuries. In model 1, the choice of initial therapeutic intervention was not associated with a statistically significant increase in risk of in-hospital mortality (adjusted OR, 1.20; 95% CI, 0.61-2.39). In addition, we used propensity-adjusted analysis. Propensity score-adjusted regression model (model 2) demonstrated no significant difference in in-hospital mortality between the laparotomy and TAE first groups (adjusted OR, 1.13; 95% CI, 0.63-2.01). The area under the receiver operating characteristic (ROC) curve, or c-statistic, of our study was 0.80, which indicated good predictive power and confirmed that the variables we selected in our propensity model were highly predictive of the treatment. We also found that age ≥ 65 years (OR, 6.24), SBP < 65 mmHg (OR, 3.33), GCS < 9 (OR, 4.97), pelvic AIS (OR, 1.54) and abdominal AIS (OR, 1.36) were independent predictors of in-hospital death for all participants. A multiple imputation approach using chained equations to account for missing covariates (age: 1 case, SBP: 3 cases, GCS: 12 cases) demonstrated similar results to those obtained on complete set analysis.

**Table 3:** Multivariable analysis of in-hospital mortality for all patients

**Variable**		**Model 1 **^**†**^		**Model 2 **^**‡**^	
		**Adjusted OR**	**95% CI**	**Adjusted OR**	**95% CI**
First procedure					
	Laparotomy	1.20	(0.61-2.39)	1.13	(0.63-2.01)
	TAE	Reference		Reference	
Age	(years)				
	≤ 25	Reference			
	26-49	1.54	(0.61-3.90)		
	50-64	2.27	(0.86-6.02)		
	≥ 65	6.24	(2.40-16.2*)		
Gender					
	male	1.12	(0.59-2.11)		
	female	Reference			
No. of comorbidities					
	0	Reference			
	1	1.00	(0.50-1.92)		
	≥ 2	2.01	(0.69-5.87)		
SBP					
	≤ 65	3.33	(1.51-7.32*)		
	66-89	1.59	(0.82-3.30)		
	≥ 90	Reference			
GCS score					
	< 9	4.97	(2.26-11.0*)		
	9-13	1.59	(0.78-3.25)		
	> 13	Reference			
ISS					
	< 26	Reference			
	26-35	1.72	(0.57-5.15)		
	36-45	1.04	(0.27-4.09)		
	> 45	1.24	(0.21-7.47)		
AIS					
	Pelvic AIS	1.54	(1.04-2.28*)		
	Head AIS	1.06	(0.85-1.33)		
	Thorax AIS	1.16	(0.91-1.47)		
	Abdomen AIS	1.36	(1.06-1.75*)		

AIS, abbreviated injury scale; CI, confidence intervals; GCS, Glasgow coma scale; ISS, injury severity score; OR, odds ratio; SBP, systolic blood pressure; TAE, transcatheter arterial embolization.

* p values significant at (p < 0.05).

† Model 1: multiple logistic regression analysis.

‡ Model 2: multiple logistic regression analysis including propensity scores; propensity scores were calculated based on age, gender, no. of comorbidities, SBP, GCS, ISS, and AIS (pelvic, head, thorax, abdomen).

The results of subgroup analyses according to SBP, pelvic AIS and abdominal AIS are shown in Table 4. Based on univariable analyses, laparotomy as the initial therapeutic intervention was associated with a significantly increased risk of in-hospital mortality (crude OR, 2.68; 95% CI, 1.32-5.44) when patients were grouped by severe pelvic AIS (pelvic AIS ≥ 4). On the other hand, multivariable analysis using logistic regression model 2 indicated that the choice of initial therapeutic intervention was not associated with a statistically significant increase in the risk of in-hospital mortality in all-patient subsets. In the limited subgroup of hypotensive patients (SBP < 65 mmHg and SBP 66–89 mmHg subgroup), the association between initial therapeutic intervention and in-hospital mortality was similar (AOR, 1.50; 95% CI, 0.56-4.05 and AOR, 1.05; 95% CI, 0.44-3.03, respectively).

**Table 4:** Subgroup analysis: association between primary intervention (Laparotomy/TAE) and in-hospital mortality

**Subgroup**		**Crude OR**	**95% CI**	**Adjusted OR **^**†**^	**95% CI**
SBP					
	≤ 65	1.50	(0.52-4.42)	1.05	(0.44-3.03)
	66-89	1.54	(0.61-3.92)	1.50	(0.56-4.05)
	≥ 90	1.52	(0.59-3.74)	1.15	(0.44-3.03)
Pelvic AIS					
	≤3	1.64	(0.70-3.84)	0.73	(0.41-1.86)
	≥ 4	2.68	(1.32-5.44*)	1.84	(0.85-3.98)
Abdomen AIS					
	≤3	1.22	(0.58-2.47)	0.87	(0.41-1.86)
	≥ 4	2.37	(0.88-6.70)	2.10	(0.78-5.66)

AIS, abbreviated injury scale; CI, confidence intervals; OR, odds ratio; SBP, systolic blood pressure; TAE, transcatheter arterial embolization.

*p values significant at (p < 0.05).

†Adjusted OR: multiple logistic regression analysis including propensity scores; propensity scores were calculated based on age, gender, no. of comorbidities, SBP, GCS, ISS, and AIS (pelvic, head, thorax, abdomen).

## Discussion

In this large, nationwide observational study, we described a unique practice pattern in Japan for multiple trauma patients with pelvic fractures and hemoperitoneum. Particularly remarkable was the fact that half (50%) of the patients who were hypotensive (SBP < 90 mmHg) in the ED underwent TAE as the initial therapeutic intervention, which is quite different from the United States and Europe. Among all patients and injury patterns, laparotomy was chosen when patients presented with more severe injuries of the whole body, especially severe abdominal organ injuries. TAE, on the other hand, was selected when patients had more severe pelvic fractures, regardless of the hemodynamic status. This descriptive analysis does confirm the soundness of the clinical judgment of Japanese surgeons who take care of critically injured trauma patients with pelvic injuries. We also noted that the choice of initial therapeutic intervention was not associated with an increased risk of in-hospital mortality. The results were similar in the subgroup of hypotensive patients. Although a methodological limitation of using FAST as a selection criterion is the lack of specific information in the JTDB describing the quantity of hemoperitoneum, our findings suggest that the choice of TAE as the initial therapeutic intervention is acceptable in some limited patients, regardless of hemodynamic status and even in the presence of proven intraperitoneal bleeding.

In this study, we could not find any evidence to support the recommendation from practice guidelines, which state that immediate exploratory laparotomy should be performed in patients who are hemodynamically unstable. The fact that TAE was the primary intervention of choice in some patients who presented with hypotension suggests that the major cause of shock was retroperitoneal bleeding from a pelvic fracture. The precision with which fracture pattern alone or hemodynamic stability alone can predict the necessity of angiography is limited [5,8,14-17].

In general, the indications for exploratory laparotomy between trauma patients both with and without pelvic fractures are identical [5,6]. In our experience, however, some patients with pelvic fractures have associated oozing from the mesentery or a retroperitoneal hematoma that passes into the abdominal cavity [1]. It is difficult to distinguish between oozing from these sites and bleeding from additional intra-peritoneal organs. We also know that a large number of patients with solid organ injuries, such as those of the liver and spleen, secondary to blunt trauma, are currently managed non-surgically [18-20]. An experimental study showed that laparotomy resulted in both a marked reduction in retroperitoneal pressure and a decreased tamponade effect in cadaveric specimens with pelvic fractures. This is due to the fact that anatomically, the pelvic retroperitoneum communicates with the space of the abdomen [21].

A previous study from the United States, which included patients who had a combination of unstable fracture pattern of the pelvis, persistent shock and abdominal injury, reported that 84% (21/25) of patients underwent laparotomy first [7]. Moreover, a previous study from Germany, which included patients who had a combination of unstable fracture pattern of the pelvis, hypotension in the ED and positive FAST results, also reported that 100% (15/15) of patients underwent laparotomy first [1]. These data suggest that it is difficult to examine the impact of initial therapeutic intervention on the outcome in pelvic trauma patients because of uniform clinical practices in the United States and Europe.

Our study is noteworthy for several reasons. First, practice variations in Japan made it possible to statistically compare the mortality between the laparotomy first and TAE first groups. Second, the JTDB contained data about FAST. As a result, we could extract many eligible patients from a nationwide database, allowing for generalization of these results. Third, we re-examined the treatment effects after we divided subjects into three subgroups according to SBP in the ED, i.e. normotension (≥90 mmHg), mild hypotension (66–89 mmHg) and severe hypotension (≤65 mmHg). Therefore, we could evaluate the outcome after adjusting the most relevant confounding effect, hemodynamic status. Finally, to evaluate the robustness of our analytical methods, we used two statistical methodologies, including the propensity score methodology. Similar results using both methods support this robustness.

Conversely, several limitations of the present study warrant mention. First, this cohort study suffers from potential residual confounders, information about which was not available within the database used. The JTDB does not record information regarding fracture patterns of the pelvis, distribution and quantity of free intraperitoneal fluid, volume and type of intravenous fluid administration and subsequent blood pressure. Thus, since hypotension was only documented on arrival to the ED, we were unable to determine whether patients were responders or non-responders to fluid replacement therapy. Furthermore, the JTDB does not record procedural findings, or whether the procedure was therapeutic or non-therapeutic. Therefore, we were unable to perform subgroup analysis according to the correctness or incorrectness of each procedure. Second, there is a potential selection bias in our study because only patients presenting to emergency hospitals participating in the JTDB were eligible for inclusion in this study. However, the institutions registered with the JTDB are not limited to Level 1 trauma centers. A previous study using the JTDB found no major differences between their data and those from the American College of Surgeons National Trauma Data Bank with regard to the characteristics of patients, and some previously published survival prediction models using the JTDB were compatible with those predicted by other national databases [11,22]. Hence, we believe that generalization of our findings is not likely to have skewed our results. Third, there is also a selection bias about the sensitivity and specificity of FAST. FAST has been shown to be an accurate diagnostic test in the setting of pelvic fractures, detecting hemoperitoneum with a specificity of 87% to 100% and sensitivity of 75% to 81% [1,5,23]. Finally, the trauma registry suffers from a number of missing data, especially unknown hospital discharge disposition. However, the proportion of missing outcomes, which we assumed to be completely random, was only 8%, and thus we do not believe it affected the direction of the observed associations.

Emergent retroperitoneal pelvic packing (PPP) for controlling life-threatening hemorrhage from pelvic fractures, although used widely in Europe and some trauma centers in the United States [24-26], has not yet been adopted in Japan. Emergent PPP seems to have some advantages in controlling hemorrhage, particularly when angiography is unavailable or would result in significant delay. However, in nearly every trauma center in Japan, the CT scanner and angiography suite are located right next to or inside the resuscitation area for trauma patients, which is directly accessible from the ED [8,27]. These trauma systems have been implemented to minimize the time from the patient’s arrival to the ED to completion of diagnosis and subsequent performance of the angiographic procedure by trauma surgeons or radiologists. Based on these factors, we suppose that there are an extremely small number of patients who underwent PPP first for controlling hemorrhage from pelvic fractures between 2004 and 2010 in Japan [8,27]. Conversely, these unique practice patterns in Japan made it possible to compare the mortality between laparotomy first and TAE first groups without the confounding influence of PPP.

Despite possible methodological limitations, our data have several important clinical implications for trauma specialists. This research, which presents comprehensive data from Japan, highlights the differences between the degree of trauma severity, trauma patterns, and practice patterns in Japan and those in both the United States and Europe. A simple comparison with other countries is not possible; however, information from a significant number of cases obtained from a nationwide survey in Japan, and the fact that Japan-specific practice patterns and their outcomes could be presented, should be viewed as extremely significant and relevant. In Japan, initial therapeutic intervention is chosen according to the severity of injuries, especially severity of abdominal organ injury and pelvic fractures, regardless of hemodynamic stability. After adjusting for confounders, including severity of injuries, the choice of initial therapeutic intervention was not associated with a statistically significant increase in risk of in-hospital mortality. In particular, it is remarkable that half of the patients who were hypotensive in the ED underwent TAE as the initial therapeutic intervention without a significant increase in the risk of in-hospital mortality. We do accept the clinical fact that decision-making in the ED involves complex assessment of multiple variables, and is not easily covered with a simple practice guideline that recommends only one approach. However, our observational study does have an important role to play in situations where randomized clinical trials are not available, to quantify procedure/treatment effectiveness and real world experiences. Even though several points need to be considered when interpreting the present findings, we believe that our study adds a small piece of evidence to the clinically relevant question of appropriate initial intervention in pelvic injury patients with hemoperitoneum. We hope our study will stimulate future multi-institutional prospective cohort studies, ideally with randomized trials, for assessing the unmeasured information that is currently unavailable in the JTDB, for improving the evidence base supporting guideline recommendations.

## Conclusions

In this large, nationwide observational study in Japan, initial therapeutic intervention is chosen according to the severity of injuries, especially severity of abdominal organ injury and pelvic fractures, regardless of hemodynamic stability. The choice of initial therapeutic intervention (laparotomy first versus TAE first) was not associated with an increased risk of in-hospital mortality.

## Abbreviations

TAE: Transcatheter arterial embolization; FAST: Focused assessment with sonography in trauma; SBP: Systolic blood pressure; AIS: Abbreviated injury scale; ISS: Injury severity score; RTS: Revised trauma score; Ps: Probability of survival; TRISS: Trauma and the injury severity score; GCS: Glasgow coma scale; JTDB: Japan trauma data bank; RR: Risk ratio; OR: Odds ratio; AOR: Adjusted odds ratio; CI: Confidence interval; SD: Standard deviation; DOA: Dead on arrival.

## Competing interests

The authors declare that they have no competing interests.

## Authors’ contributions

All authors participated in the development of this project. MK, SY, SF, KM, and SF designed this study. MK, and TY. contributed to the acquisition of data; analysis and interpretation of data were completed by MK, SY, SF, and SF The manuscript was drafted by MK, SY, SF, and SF; and critical revision of the manuscript was undertaken by MK, SY, SF, and SF. All authors read and approved the final manuscript.
